# Manoeuvring Through the Crisis: Labour Market and Social Policies During the COVID-19 Pandemic

**DOI:** 10.1007/s10272-020-0937-6

**Published:** 2020-12-01

**Authors:** Werner Eichhorst, Paul Marx, Ulf Rinne

**Affiliations:** 1 IZA Institute of Labour Economics, Schaumburg-Lippe-Str. 7-9, 53113 Bonn, Germany; 2 Institute for Socio-Economics, University of Duisburg-Essen, Lotharstr. 65, 47057 Duisburg, Germany

## Abstract

The unprecedented COVID-19 pandemic has a severe impact on societies, economies and labour markets. However, not all countries, socio-economic groups and sectors are equally affected. Part of this disparity can be related to the different role and extent of short-time work, which is now being used more widely than during the Great Recession. Furthermore, unemployment benefits have been made more generous in many countries. While it is still too early to assess the relative success of national strategies to cope with the pandemic and to revitalise the labour market as well as to evaluate the medium-term fiscal viability of different support measures, a few policy directions become apparent. These include the use of digital tools to increase resilience against economic shocks, the longer-term perspective of short-time workers in the current crisis, social protection for self-employed workers that is robust to economic crises and resilient models for school-to-work transitions of younger workers.

Economic and social disruptions caused by the coronavirus pandemic may have lasting effects on employment, income and working conditions. At the same time, there are significant cross-country differences in the labour market and social policy responses that are deployed to help mitigate the direct effects of the crisis. This article is based on the ongoing Institute for the Study of Labor (IZA) Crisis Response Monitoring covering a sample of European countries and the US.[1] It summarises major changes in labour markets and provides an assessment of crisis-related interventions. It also points to policy issues that are likely to emerge in the near future.

* The authors wish to thank Patrick Arni, René Böheim, Thomas Leoni, Pierre Cahuc, Tommaso Colussi, Rui Costa, Stephen Machin, Priscila Ferreira, João Cerejeira, Miguel Portela, Lena Hensvik, Oskar Nordström Skans, Susan Houseman, Egbert Jongen, Paul Verstraten, Martin Kahanec, Monika Martišková and Raul Ramos for their invaluable effort and contributions to the IZA Crisis Response Monitoring in these strange and challenging times.

**Ulf Rinne**, IZA - Institute of Labor Economics, Bonn, Germany.

## Unequal labour market impacts of COVID-19

Early forecasts about the economic impact of COVID-19 were clearly too optimistic. Over the past months, there has been a progressive deterioration of forecasts and of the actual economic situation. The latest forecasts by the OECD Interim Economic Outlook from September (OECD, 2020a) expect strong declines of GDP between 4% and 8% in 2020. Quite negative scenarios of about -10% apply to countries with severe and protracted lockdown phases such as Italy or France, but also to the UK. However, the labour market impact is likely to also differ depending on the specific institutional arrangements, employment structures and crisis response measures.

Looking at the development of unemployment over the course of 2020, there has been a massive increase in countries like Spain and Sweden. This increase has been even larger in the US, while other countries saw a rather moderate reaction of unemployment, e.g. France, the UK or Italy. While this might be due to some delays in data re-

1 More specifically, the IZA Crisis Response Monitoring (https://covid-19.iza.org/crisis-monitor) includes the following sample of countries: Austria, France, Germany, Italy, the Netherlands, Portugal, Slovakia, Spain, Sweden, Switzerland, the UK and the US.

Figure 1
**Unemployment and short-time work, 2020**

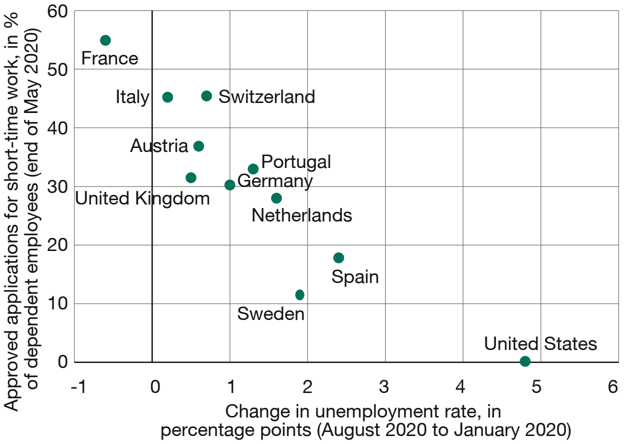

Notes: Regarding short-time work, some countries only provide notifications that are not necessarily identical with the number of individuals actually taking up short-time work at a later stage. Switzerland and UK: data for unemployment rates are based on national databases; end point for UK is July 2020.

Sources: OECD.Stat, Monthly Unemployment Rates; OECD Employment Outlook 2020, Figure 1.8; amstat.ch; ons.gov.uk.

porting, institutional explanations arguably matter more. Some countries have seen a massive decline in working hours in general, and in particular a massive increase in notifications for and take-up of short-time work (although there is a lack of precise data). Hence, countries can be mapped along the following two dimensions: the increase in unemployment and the extent to which short-time work is used, based on broadly comparable data available.

Figure 1 provides the general picture, which points towards an inverse relationship between the expansion of short-time work and changes in unemployment. The US is the most prominent case of a steep unemployment increase. To date, many other countries see a relatively stable development of unemployment (and employment). This is partly caused by declining labour force participation (e.g. Italy), but mostly by a steep increase in short-time work in the first half of 2020. Germany, for example, which successfully used short-time work during the Great Recession (Brenke et al., 2013), shows a current take-up that is significantly higher than during the 2008-09 crisis. In the second quarter of 2020, a peak of about six million short-time workers (about 20% of dependent employees) was reached. The number fell to less than four million in September (ifo, 2020).

While the current crisis affects virtually all sectors to some extent, albeit in different ways, its impact is highly unequal across socio-economic groups. Even in countries with moderate overall unemployment reactions, not all sectors use external flexibility (dismissals, termination of fixed-term contracts, reduction of temporary agency work) and internal flexibility (short-time work, work sharing) to the same extent. This strongly depends on the willingness of employers to "hoard" certain types of skilled labour that is hard to replace, as well as on employment protection legislation.

When studying individual country experiences more closely, employment losses tend to be concentrated in sectors that were directly affected by lockdown measures, disrupted value chains or general economic uncertainty. In fact, the sectoral composition of jobs destroyed appears quite similar across countries. Most affected by declines in working hours and employment were the hospitality sector, leisure and tourism, cultural activities and events, local retail trade and logistics. Employment in manufacturing declined in some countries only. At the same time, temporary peaks in demand could be observed in the health sector, supermarkets, online retail trade and delivery services.

Furthermore, particular difficulties are clearly visible for low-skilled workers (and migrants), given the sectoral composition of their jobs and their limited ability to work from home. The latter has critically contributed to the continuation of work in many white collar jobs. At the same time, many young labour market entrants, and jobseekers in general, suffer from a massive decline in vacancies and hiring rates. In Continental Europe, the crisis has so far reduced hiring rates more than it increased dismissal rates. Against this background, job transitions for school leavers and apprentices will be hampered.

The unequal labour market impacts of COVID-19 become particularly apparent for persons with temporary or variable contracts. For example, fixed-term employment is declining more strongly than permanent contracts in countries where this divide is particularly relevant, such as Spain, Portugal or France. Temporary agency workers face a larger risk of being made redundant (e.g. in the Netherlands or Slovakia). The same holds true for marginal part-time workers, on-call workers and independent contractors in sectors that are heavily affected, despite some efforts to incorporate them better into social protection and short-time work schemes (e.g. in Switzerland or the Netherlands).

The actual effect of the crisis on specific categories of workers, however, also depends on the institutional setting in the respective country and on the sectoral or occupational composition of non-standard work. In many cases, those affected by labour market disruptions have no or only very limited access to social insurance, and thus heavily depend on means-tested income support and ad hoc relief measures.

Two interrelated areas aiming at stabilising jobs and income are of particular importance in the early phase of countries' crisis responses: support to small firms and self-employment workers and as well as short-time work and unemployment benefits.

### Supporting self-employed individuals and small firms

The situation of small businesses has been a particular concern for policymakers in many countries. Several countries, e.g. Austria, France and Germany, have set up funds from which lump-sum payments to small businesses are financed. Sometimes tax reductions (e.g. in Sweden) or specific loans (e.g. in the US, Italy and Sweden) were reserved for small companies.

A second, larger concern has been the social security of self-employed individuals with no or few employees, who are usually poorly integrated in unemployment insurance. Already over the past decade, a debate has emerged about whether European employment models depend excessively on low-paid and precarious forms of self-employment (Eichhorst et al., 2013; Boeri et al., 2020). The pressure to act, resulting from a larger number of unprotected jobless individuals who were previously self-employed, forced many countries to adjust their social security systems in the current crisis. The general trend is to provide benefits roughly at the level of social assistance, but with more lenient access and behavioural requirements. The variety of measures can be illustrated with some examples.

The Netherlands created a temporary benefit scheme for three months on the municipal level that provides benefits (at the level of social assistance) for the self-employed without strict means-testing (called *Tozo*). Until May 2020, *Tozo* was claimed by almost a quarter of all self-employed workers in the Netherlands. The scheme was recently extended until mid-2021, but now includes a partner's income test. Italy temporarily pays a monthly allowance of €600 to self-employed and other non-insured types of workers. In the UK, the new Coronavirus Self-Employed Income Support Scheme provides taxable grants corresponding to 80% of the average monthly trading profits of the self-employed individual (up to a total of £7,500). By mid-June 2020, already 2.6 million people had applied for these grants. A similar temporary scheme providing partial income replacement for self-employed and small firms was introduced in Sweden during the first months of the coronavirus pandemic (until July).

In most countries, the measures taken to protect the self-employed were implemented ad hoc and adopted temporarily. From this perspective, it is quite remarkable that Spain used the crisis as an opportunity to permanently modernise its social assistance, which previously differed across municipalities. Since June 2020, there is a unified system (*Ingreso Minimo Vital*) that pays up to €1,000 to families and €460 to one-person households who fall in the definition of severe poverty (below 40% of median income). This will make the social security net much more consistent. In addition to this new system, Spain also provides a temporary subsidy to the self-employed.

In sum, there is a variety of measures to support the liquidity of small companies and to stabilise the income of the self-employed. Many of the schemes were rather improvised, and it is not clear to date whether the implementation has been sufficient to provide rapid assistance. In Italy, for instance, the Central Guarantee Fund provides loans up to €2,500 to small and medium-sized enterprises (SMEs) that are fully guaranteed by the Italian state. According to preliminary research, only a minority of eligible firms have applied, presumably because of bureaucratic application procedures. In Switzerland, it has been noted that there is a gap between guaranteed loans that have been approved and that have actually been taken up by firms. A reason could be that SMEs try to avoid indebtedness. This is also related to the question of what happens if the crisis lasts longer and SMEs face difficulties repaying their state-provided or guaranteed loans. Another implementation issue has arisen in Germany with lump-sum payments to the self-employed and small businesses. During the improvised roll-out, miscommunication initially led to the widespread impression that the money may be used to cover personal expenses, whereas it actually is reserved to cover business-related expenses. The self-employed can only rely on social assistance for income replacement. It is currently unclear if and to what extent the state will reclaim misallocated payments. Also in the UK, surveys show that at least in the initial period of the Coronavirus Self-Employed Income Support Scheme, there was widespread confusion about eligibility criteria. Problems such as these illustrate that most countries have much less experience in setting up and actually implementing measures to protect self-employed compared to dependent workers.

### Short-time work and unemployment benefits

Initial crisis responses have shown some remarkable similarities across countries. Most countries have tried to use some kind of short-time work scheme to keep workers in their jobs and to maintain low unemployment. This appears reasonable because it had been expected (at least initially) that the COVID-19 crisis would follow a V-shaped pattern, also when compared to other and more common types of economic shocks. In such a situation, i.e. a sharp demand slump followed by rather quick economic recovery, short-time work schemes have their strongest justification (Cahuc, 2019).

Several countries already had such schemes in place at the beginning of the crisis. In almost all cases, additional measures were taken to expand existing schemes. The goal was usually to make them more inclusive and to lower the residual costs for employers. Austria introduced, for instance, a temporary COVID-19 short-time work scheme that is more generous than the pre-existing version, and Sweden started a completely new short-time work scheme. In Spain, France and Italy, additional sectors were included in the scheme and/or the requirements for eligibility were relaxed. Germany eased eligibility requirements for firms, raised replacement rates and opened up the possibility for temporary agency workers to benefit from the scheme. The idea to include vulnerable types of workers was pursued even more decidedly in Switzerland, where workers on fixed-term contracts, apprentices, temporary workers, on-call workers and even family members helping in small firms are also entitled, at least temporarily. In the UK, the Coronavirus Job Retention Scheme allows companies to send employees on leave, while 80% of the salary is compensated by the state. Compared to short-time work schemes in most other countries, this programme now also allows workers to take up part-time jobs in other companies (furloughing). This is an interesting institutional feature that could be considered in other countries. The Netherlands, Slovakia and Sweden have set up schemes in the crisis that closely resemble the typical parameters in other countries.

In most countries, applications for short-time work have soared in the crisis. It certainly is a feature at the core of the European crisis response. Besides the replacement rate, important institutional differences relate to the extent to which employers can reduce labour costs. This includes the remaining wage share that has to be covered and the question of whether social security contributions are waived. Some countries, such as France and Germany, allow short-time work without any costs to employers, but that is not the standard in Europe. At the other end of the spectrum is Portugal: In its temporary layoff scheme, employers still have to cover 30% of the wage, which makes the scheme considerably less generous.

Recently, however, there has been a widespread movement towards a gradual phasing out. Since 1 June 2020, for example, France has again imposed costs on employers for the use of *activité partielle* in sectors where economic activity has gradually resumed. While workers still receive 70% of their normal gross wage for unpaid hours, companies now pay 15% of this amount. As a result, the cost of employees' reduced working hours for a company has risen to 10% of the usual full-time labour cost. Sectors that are still subject to restrictions (e.g. tourism, hospitality or culture) are excluded. *Activité partielle* has become even less generous to both companies and employees since October 2020. The gross wage replacement rate for unworked hours has fallen from 70% to 60%, and companies have to pay for 40% of this, bringing the cost of an employee's unworked hours for a company to 24% of the usual full-time labour costs. At the same time, however, a new instrument has been created for companies in more protracted difficulties. Beginning on 1 July 2020, companies with longer-term difficulties will be able to apply for the *activité réduite de maintien dans l'emploi*, which is limited until 2022. The scheme provides compensation for a maximum reduction in working hours of 40% (50% in exceptional cases) and ensures that employees receive 70% of their normal gross wage for hours not worked. Employers must pay 15% of the payments that workers receive for entitlements that begin before 1 October 2020 and 20% for entitlements that begin after that date. Claims can only be made if there is an agreement between employees and employers, and the agreement may explicitly prohibit any layoffs.

The Netherlands started to reduce the generosity of the *Tijdelijke Noodmaatregel Overbrugging voor Werkbehoud* (NOW) scheme. When extending this scheme to mid-2021 recently, the subsidy was lowered from 90% to 60% while additional resources are simultaneously provided for retraining and mobility support to encourage returning to longer (usual) working hours or job-to-job changes.

In Austria, too, short-time work enters into a new phase this autumn. The Austrian 'Corona short-time work' (phase 1), which was initially limited to six months, was extended by one month to the end of September (phase 2), while the new short-time work model (phase 3) began in October and companies can apply for six months. With that switch, the minimum working time was raised from 10% to 30 %; however, it may be reduced below this minimum in special cases when social partners agree. The maximum working time is 80% now (previously it was at least 10% and the maximum was 90%). Employees have to be available for further training during the short-time work if this is offered by the company. In small companies, new offers for further training are to be created in cooperation with the Austrian public employment service.

In Sweden, the regular system of short-time work, which was only introduced at the beginning of 2020 in a more generous way than originally planned, was extended until the end of 2020. Furthermore, Swedish employers were allowed to reduce their employees' working hours by up to 80% for three months from 1 May 2020 (instead of 60% for a maximum of six months before that date), with the state bearing the bulk of the costs. In conjunction with the employers' reduced social security contributions, employer costs were reduced by up to 86% from May to July.

A less generous model will apply until the end of 2020, however, and an even more frugal regular system will apply starting in 2021.

Although the unemployment insurance system was not the primary focus of crisis responses and adjustments of European policymakers, many countries have extended eligibility or eased access. This was particularly relevant in Sweden, where the income ceiling for calculating benefits is rather low and a Ghent system with limited coverage through unemployment insurance funds applies. The government reacted, inter alia, with shortening the membership duration in funds that is necessary for eligibility to insurance benefits and with raising the benefit ceiling. Spain went into a similar direction by temporarily suspending the minimum contribution periods for unemployment insurance. Complementing the focus on keeping workers in their jobs during the crisis, some countries have temporarily changed dismissal regulation. Layoffs were banned or restricted in Italy, Portugal and Spain, to name just a few countries.

### Blind spots

Younger workers and immigrant workers may be particularly hit by the current crisis. In contrast to previous recessions, in the current situation, many sectors that offer entry-level jobs (e.g. retail, hotels and restaurants) are also affected. In some countries, it is even the case that precisely those sectors that used to absorb part of the downward pressure on employment in previous recessions are now the most affected. This considerably worsens the outlook for new labour market entrants – and also for groups such as refugee immigrants – for whom labour market conditions upon entry may have lasting negative effects (Aslund and Rooth, 2007).

The situation for younger workers may be particularly challenging in countries that heavily rely on the dual apprenticeship system as it depends on firms' demand for apprentices. This could imply that younger workers in these countries do not only face deteriorating employment prospects after their graduation, but also that a larger part of the usual vocational training system leading to degrees and certificates may come to a halt. However, as the majority of apprenticeships only started in late summer of 2020, it seems too early to judge how this situation will evolve. In any case, demand and supply on the vocational training market should be closely monitored – especially in countries with a strong dual apprenticeship system.

Other countries, mainly in Southern Europe, may also face enormous challenges with respect to new labour market entrants. Although these countries do not rely on dual apprenticeship systems, they have to deal with notoriously high youth unemployment rates. With regards to school-to-work transitions, a rational approach in the current situation appears to be a longer stay in education. However, this implies increased competition after the crisis. It may also imply the need for additional funding on an emergency basis aimed at both students and educators.

Various policy responses to best support labour market entrants are currently being considered – albeit with remarkable heterogeneity across countries. While there is an intensive policy discussion and also rather concrete initiatives in some countries, the situation of new labour market entrants has not yet received much attention in others. The latter seems to be the case in Southern Europe, but also in Switzerland and the US. In countries with rather concrete initiatives, these measures appear to strongly depend on institutional context. For example, a joint federal support initiative to make apprenticeship capacities more resilient in times of crisis came into effect in Germany. The programme includes bonus payments to SMEs that provide apprenticeships, bonus payments to firms taking over a current apprentice from a firm that has gone bankrupt during the crisis, support to avoid short-time work among apprentices and incentives for training in facilities outside individual firms. Hiring subsidies targeted at younger workers and subsidies for apprentices have been introduced in France, and further labour cost reductions to hire young people are to be implemented in Sweden.

### Necessary reforms beyond the coronavirus crisis

In many countries, one can currently observe governmental interventions at an unprecedented speed and breadth. The short-term objectives of various policy responses are predominantly income and employment stabilisation. In some countries, these responses rely to a larger extent on automatic stabilisers, while the amount of discretionary measures is generally large and often unprecedented. Yet, the degree of policy innovation appears more incremental than revolutionary – possibly with the exception of short-time work schemes. This instrument has been expanded or newly introduced in a number of countries.

It is a common perception that the current crisis accelerates structural change and digitalisation. Firms may increasingly view digital tools as a means to increase their resilience against external economic shocks. In this respect, the crisis is also an endurance test of firms' (and countries') past digital achievements. Past omissions become very visible. Other pre-crisis trends – often sector-specific – may be amplified by the crisis. For example, it appears likely that the long-term decline of local retail and smaller shops will accelerate, while all forms of online retail will experience an extra boost. In addition, the ongoing transformation of manufacturing, in particular of car manufacturers and their suppliers, may proceed even more rapidly than previously expected.

The crisis highlights old and new types of inequalities that are related to structural weaknesses in the labour market and welfare states. These inequalities call for further redesign so that institutions are in a better position to support workers and firms on the road to recovery. A few points are of particular relevance:

First, the crisis has highlighted the usefulness of digital tools in communication as well as in private and public service provision. To avoid long-term scarring and deepened inequalities, blended learning at schools and universities is essential to stabilise human capital formation not only when personal contact is restricted. Furthermore, digital tools should help restart active labour market policies and individual activation, such as job search support, placement and training. This is especially important in a situation of high unemployment (after the phasing out of short-time work) and if structural changes make a return to existing job profiles unlikely.

Second, the longer firms and workers rely heavily on (generous) short-time work schemes, the less likely becomes a full return to the 'old normal' because markets and business models change. Rather, the main challenge is to prepare for either changing job requirements when firms undergo internal restructuring or for a move to other employers, sectors or occupations if external labour market mobility is the only viable option. This makes retraining essential, ideally combined with work experience in firms, sectors or occupations with better employment prospects. It might make sense to stipulate that advice on future job options, related training and real-world work experience (e.g. via internships, posting or qualified secondary education) are mandatory elements of extended public short-time work support. In a way, this would imply a partial detachment of long-term short-time workers from their employer even when the employment relationship continues to exist. Current policies in most countries do not effectively move in that direction and neglect the training and mobility component in short-time work. Providing mostly 'passive' short-time work support for a longer period, as in Germany, is not a sustainable solution.

Third, the peculiar situation of self-employed workers has triggered the creation of ad hoc support schemes that are hardly integrated into the regular unemployment insurance system. In some cases, access to means-tested income support was facilitated by lifting requirements. However, the creation of a more long-lasting system of social protection for self-employed workers is a pending issue. One option would be to integrate people who are engaged to a substantial extent in self-employment into public unemployment insurance (and old-age insurance), at least after an initial start-up phase. By doing so, it would be possible to establish a clear link between benefits and contributions comparable to dependent workers.

Fourth, for young people leaving school or university, building bridges that establish early contacts to employers and provide work experiences remains of utmost importance. Vocational training as a joint responsibility of public actors, employers and trade unions has been one key element of resilient youth labour markets in the past. This model continues to provide relatively smooth transitions, also due to the continued commitment of stakeholders. Where it does not exist, governments and social partners should try to establish functionally equivalent programmes that help integrate young people into initial jobs along with the provision of relevant skills, after school or university. In the current situation, this will require digital tools. The idea of the European Youth Guarantee may be helpful, but calls for systematic implementation in countries and regions that are particularly affected, ensuring a sufficient quality of work and/or training offers.
